# BronchStop Study Protocol, Season 2

**DOI:** 10.3310/nihropenres.14097.2

**Published:** 2025-12-02

**Authors:** Shaun O'Hagan, Steve Cunningham, Simon B Drysdale, Helen E Groves, Samantha Hunt, Dalia Iskander, Xinxue Liu, Mark D Lyttle, Chengetai D Mpamhanga, Thomas Waterfield, Thomas C Williams, Robin Marlow, Damian Roland

**Affiliations:** 1 Queen's University Belfast Wellcome-Wolfson Institute for Experimental Medicine, Belfast, Northern Ireland, UK; 2 Department of Paediatric Respiratory and Sleep Medicine, Royal Hospital for Children and Young People, Edinburgh, UK; 3 Centre for Inflammation Research, University of Edinburgh, Edinburgh, UK; 4 University of Oxford Oxford Vaccine Group, Oxford, England, UK; 5 NIHR Oxford Biomedical Research Centre, Oxford, England, UK; 6 Paediatric Emergency Medicine Leicester Academic (PEMLA) Group, Leicester Royal Infirmary, Leicester, UK; 7 Department of Anthropology, University College London, London, England, UK; 8 Research in Emergency Care Avon Collaborative Hub (REACH), University of West of England, Bristol, UK; 9 Emergency Department, Perth Children's Hospital, Perth, Western Australia, Australia; 10 Child Life and Health, University of Edinburgh, Edinburgh, Lothian, EH16 4TJ, UK; 11 Emergency Department, Bristol Royal Hospital for Children, Bristol, UK; 12 University of Bristol, NIHR Health Protection Research Unit in Behavioural Science and Evaluation, Bristol, UK; 13 Sapphire Group, Population Health Sciences, University of Leicester, Leicester, UK

**Keywords:** Respiratory syncytial virus; maternal vaccination; respiratory disease; infants

## Abstract

**Introduction:**

In 2021 we launched the BronchStart study, which collected information on 17,899 hospital attendances in children with serious respiratory tract infections following the release of lockdown restrictions. Our study informed the Joint Committee on Vaccination and Immunisation’s (JCVI) decision to recommend the introduction of maternal respiratory syncytial virus (RSV) vaccination, which was rolled out in the United Kingdom in August/September 2024 for all pregnant women at a gestation of 28 weeks or more. That winter we performed the BronchStop study, which examined vaccine effectiveness in its first season, conducted a survey of mothers to understand factors affecting vaccine uptake, and collected RSV positive samples for molecular epidemiology.

**Methods and analysis:**

In the winter season of 2025-2026 we will conduct a UK-wide, multi-centre, prospective, test-negative case control study. The aim is to assess the effectiveness of maternal RSV vaccination against hospitalisation for RSV-associated acute lower respiratory tract infection (ALRI) amongst infants under the age of 6 months born to vaccine-eligible pregnant mothers. A survey designed in partnership with our public and patient involvement (PPI) group will be administered to mothers of recruited infants to understand factors affecting maternal vaccine uptake. RSV-positive samples will undergo whole genome sequencing, and all samples will undergo real-time, reverse transcriptase polymerase chain reaction (rRT-PCR) testing for a panel of respiratory viruses to understand residual causes of severe infant respiratory disease in the post-vaccination era.

**Ethics and dissemination:**

Participants recruited to the study will be asked for informed consent to participate in the maternal survey, for researchers to access their vaccination records, and for routinely collected virological samples from their infants to undergo rRT-PCR testing. Regular reports to advisory groups, including JCVI and the World Health Organisation, and for peer-reviewed publications are planned to disseminate findings and inform decision-making.

## Plain Language Summary

**Background to the research:**

Respiratory syncytial virus (RSV) is a common virus that can cause serious breathing problems in babies, sometimes leading to hospital admission. Our initial BronchStart study played an important role in helping the UK government to decide to offer a new RSV vaccine in pregnancy, which protects newborn babies. In August/September 2024, as the new vaccine was introduced in the UK, we conducted the BronchStop study to understand how well the vaccine worked during its first winter.

**Aim of the research:**

We now want to study how well the RSV vaccine in pregnancy protects babies during its second winter of use, when it has become part of routine NHS care. Our research will:

- Measure how effective the vaccine is
- Explore why some women choose to have the vaccine, while others don’t
- Monitor whether the virus is changing in ways that could affect vaccine effectiveness
- Identify other viruses causing breathing problems in babies

**Our research plan:**

We will carry out a UK-wide study during the 2025–2026 winter season to see how the vaccine is working in real-life conditions. Our study will focus on babies under six months who are admitted to hospital with breathing problems, and check whether they test positive for RSV or other common viruses. We’ll invite mothers to complete a survey, co-designed with parents and carers, to better understand their views and experiences of the vaccine.

**Patient and Public Involvement (PPI):**

Our Public and Patient Involvement (PPI) group helped us to design the survey and provided feedback to make sure our research is parent and carer-friendly and relevant. As the study continues, we will keep working closely with these groups to guide our approach and to ensure we share results in ways that are clear and meaningful for families.

## Introduction

### Background and rationale

The BronchStart study was launched in 2021 ^1– 3^, anticipating an increase in serious respiratory infections in children following the release of lockdown measures implemented to limit the spread of SARS-CoV-2 during the COVID-19 pandemic. This study documented serious early-life respiratory disease in the United Kingdom, collecting detailed information on respiratory admissions in infants and children, and informed the Joint Committee for Vaccination and Immunisation (JCVI) decision to introduce widespread RSV immunisation to the UK ^4^. Maternal RSV vaccination (RSVpreF) was rolled out from August 12, 2024 (Scotland ^5^) and September 1, 2024 (England ^6^, Wales ^7^ and Northern Ireland ^8^), offered free-of-charge for pregnant individuals at a gestation of 28 weeks or more. In the first season of maternal vaccine roll-out, the BronchStop study (the continuation of BronchStart) provided vaccine effectiveness (VE) estimates for preventing RSV-associated infant hospitalisation ^9^, surveyed mothers of infants admitted with respiratory disease to understand factors affecting vaccine uptake ^10^ and conducted molecular epidemiology studies to examine whether VE was affected by RSV type. Preliminary results were shared with the JCVI and the World Health Organization (WHO) and informed the WHO’s decision to recommend immunisation to protect infants against RSV disease globally ^11^.

Despite being a highly effective intervention, RSVpreF vaccine coverage in the United Kingdom remains low (54.7% at the latest estimate in England ^12^ and 49.6% in Scotland ^13^), in keeping with vaccination trends globally ^14^. Additionally, whilst our preliminary estimates show high VE against RSV-associated acute lower respiratory tract infection (ALRI) admission in infants whose mothers were vaccinated more than 14 days before delivery (72%, 95% CI: 48–86) ^15^, this VE analysis was limited to the youngest age group (the oldest infant recruited to the study was 5 months of age and the median age of cases was 1.6 months). We could also not assess the effectiveness of the programme once it reached a steady state, where the majority of pregnant individuals receive the vaccine at 28 weeks’ gestation. Additionally, whilst some infants were tested for viruses other than RSV, we were unable to ascertain what the main viral causes of ALRI are in the infants of vaccinated and unvaccinated mothers, to guide the development of further interventions to reduce the burden of severe respiratory disease in this population.

In July 2025 the governments of the four nations of the United Kingdom announced that nirsevimab, a long-acting monoclonal antibody that protects against RSV infection, will be offered to infants at high-risk of severe RSV disease (those with chronic lung disease of prematurity, requiring home oxygen or long-term ventilation, haemodynamically significant congenital cardiac disease, or severe combined immunodeficiency) ^16^. Replacing palivizumab, the eligible population will be expanded to include all infants born at <32 weeks' gestation - infants who would be less likely to benefit from maternal RSVpreF vaccination. The BronchStop research infrastructure will also allow us to evaluate reasons for incomplete nirsevimab uptake, should coverage be lower than anticipated, and understand reasons for incomplete nirsevimab protection in this relatively poorly studied group of high risk infants ^17– 19^.

As the maternal RSVpreF vaccination programme in the United Kingdom enters a steady state - one where most pregnant individuals receive the vaccine at, or close to, 28 weeks- we will conduct the study over a second RSV season to understand VE of the RSVpreF vaccination programme; evaluating protection for older infants, maternal factors affecting RSVpreF and nirsevimab uptake, and viral molecular epidemiology in the post-immunisation era.

## Protocol

This protocol is structured in keeping with the principles of the STROBE statement.

### Patient and Public Involvement

Previous work with a patient and public involvement (PPI) group highlighted that mothers would be happy for their vaccination records to be accessed to ascertain their RSVpreF and pertussis immunisation status, if they were able to provide informed consent for this. For the second season of the study, we engaged a PPI group of recent mothers to obtain feedback on specific aspects of the study design. The group were asked whether they would be willing for routinely collected virology samples to be tested for non-RSV viruses, whether they believed most mothers would be able to accurately recall their estimated due date (EDD), to enable precise calculation of gestational age at RSVpreF administration and at birth, and whether they had any preferences for inclusive and respectful language when referring to individuals who are pregnant or have recently given birth in future study publications. All participants confirmed they would be happy for routinely collected nose/throat samples to be tested for non-RSV viruses. They also expressed confidence that most mothers would be able to accurately recall their estimated due date (EDD). Regarding preferred terminology for future publications, participants were presented with a pre-specified list and could select multiple options. Responses included: “pregnant women” (72%), “mothers” (29%), “pregnant individuals” (14%), “birthing parent” (14%) and “no preference” (14%).

### Study design

We will conduct a national multi-centre test-negative case control study to assess the effectiveness of maternal RSVpreF vaccination against hospitalisation for RSV-associated ALRI amongst infants born to vaccine-eligible pregnant mothers. A test-negative design was chosen as this reduces the chance of bias, including collider bias ^20^ due to differential healthcare seeking behaviours ^21^.

### Study objectives

Primary objective:

- To calculate the VE of RSVpreF when administered in a steady state vaccination programme in protecting against hospitalisation with RSV-positive ALRI in infants <6 months of age

Secondary objectives:

- Calculate the VE of RSVpreF when administered more than 14 days before delivery in protecting against RSV hospitalisation in infants < 6 months of age
- Calculate the overall effectiveness of the UK’s combined RSV immunisation programme in protecting against RSV hospitalisation in infants <6 months of age
- Explore how the VE of RSVpreF in protecting against RSV hospitalisation is affected by infant age and maternal gestation at vaccination.
- Describe levels of care and respiratory support in RSV-positive infants of mothers vaccinated more than 14 days prior to delivery, compared to infants of unvaccinated mothers, including hours of invasive mechanical ventilation if ventilated
- Describe facilitators and barriers to maternal vaccine and nirsevimab uptake, and analyse whether those for maternal vaccination have changed between the first and second season of RSVpreF administration
- Conduct rRT-PCR testing to understand non-RSV causes of ALRI in the infants of RSVpreF vaccinated and unvaccinated mothers

### Participants and recruitment

***Population and eligibility***

**Inclusion criteria**

Eligible participants will be infants aged 6 months or less at the time of admission to a hospital with clinical features of bronchiolitis (cough, tachypnoea or chest recession, and wheeze or crackles on chest auscultation ^22^, lower respiratory tract infection (clinical diagnosis) or a first episode of acute viral wheeze, as per the original BronchStart protocol ^1^.

Eligible infants will need to have undergone real-time reverse transcriptase polymerase chain reaction (rRT-PCR) or equivalent testing for RSV during their admission. For the primary outcome, inclusion criteria will include birth at greater than 28 weeks of gestation (as assessed by antenatal ultrasound scanning earlier in the pregnancy).

We will recruit infants aged less than 6 months because passive immunity conferred through maternal vaccination is highest early in life and wanes thereafter, with limited data supporting sustained protection in infants beyond 6 months of life ^23, 24^. Additionally, including older infants (up to 12 months) would require a substantially larger sample size to maintain the desired precision for the maternal VE estimate, exceeding the anticipated recruitment capacity of this study.

**Exclusion criteria**

Infants older than 6 months at the time of admission, infants not admitted to a hospital ward from the Emergency Department (ED), and infants not tested for RSV using rRT-PCR or equivalent during their admission will be excluded. For the primary outcome, additional exclusion criteria will be receipt of nirsevimab and birth at a gestation of less than 28 weeks.

***Data collection***

We will collect data at two time points: baseline (date of presentation to a participating hospital) and seven days later. Informed consent will be sought from mothers of eligible infants for permission to conduct a questionnaire, and access maternal medical records for their RSVpreF and pertussis vaccination status, and infants for their nirsevimab immunisation status. Clinicians identifying a case for inclusion will keep a local log of participants. An email after 7 days to the submitting researcher will prompt data entry at this point.

**Variables to be measured**

At baseline, data including patient demographics, presenting characteristics, acuity and results from routine point-of-care virology testing will be collected (see **Supplementary File 1**, also available at http://dx.doi.org/10.7488/era/885). At seven days data collection will include the infant’s length of stay (if this is longer than seven days, further reminder emails will be sent on a weekly basis), highest acuity dependency (the ward they were placed on if admitted: Observation Unit, Normal, High Dependency or Intensive Care), whether care the patient was discharged or died and (if obtained) what viruses were identified by rRT-PCR (see **Supplementary File 1**). An external link will enable researchers to enter a full postcode-derived index of multiple deprivation (IMD, for England), a Scottish IMD (SIMD), a Welsh IMD (WIMD) or a Northern Ireland Multiple Deprivation Measure (NIMDM) for database entry. If a patient received invasive mechanical ventilation (IMV), the total number of hours of IMV received will be recorded.

RSV status will be identified by nasopharyngeal aspirate/swab (NPA/NPS) tested by either (a) point of care testing (rapid viral testing where available) at baseline presentation to the Emergency Department (ED), or (b) by laboratory rRT-PCR testing, performed as part of standard care in participating paediatric centres ^15^.

**Participant questionnaire**

The mothers of infant participants will be asked self-defined gender, self-defined ethnicity (five groups as per the UK census ^25^), whether they are currently breastfeeding, the estimated due date of the pregnancy, and infant gestation at birth.

They will also be asked for their views on the maternal RSVpreF vaccination. The questionnaire used in the first season of the BronchStop study was designed in keeping with the 5Cs of vaccine hesitancy (confidence, complacency, constraints, calculation, collective responsibility). In the first season of utilisation we found that whilst the questions regarding vaccine confidence, complacency and constraints were easily understood by respondents, the questions regarding calculation (“When I consider the maternal RSV vaccine, I weight its benefits and risks to make the best decision possible”) and collective responsibility (“When everyone has the maternal RSV vaccine, I don’t have to be vaccinated too”) were less well understood and sometimes led to confusion. For the second season we have therefore shortened this part of the questionnaire to reduce load on participants and researchers, removing the questions on calculation and collective responsibility.

Additionally, mothers completing the participant questionnaire will be asked whether they are willing to be contacted about participating in an in-depth interview for a proposed qualitative study (currently under ethical review), titled *“Understanding Parental Decision-Making in RSV Prevention: The BronchStop Qualitative Study.”* This study will explore and interpret parental attitudes towards the maternal RSVpreF (Abrysvo®) vaccine in pregnancy and the infant long-acting monoclonal antibody, nirsevimab (Beyfortus®), in order to identify perceived barriers and facilitators to uptake of both preventatives. As well as interviewing mothers, we will conduct purposive sampling to recruit parents and caregivers through community channels to ensure a broad range of perspectives are captured.The findings of this study will inform the development of tailored antenatal education and counselling resources, as well as supporting ongoing vaccine implementation efforts across the UK.

***Exposure and outcomes***

The primary outcome will be infant hospitalisation with RSV-associated ALRI. The treatment exposure will be maternal RSVpreF receipt status prior to birth amongst both case and control patients. To ascertain this, maternal medical records will be accessed either locally by research staff, by searching secondary care maternal records or contacting the participant’s primary care physician, or centrally by a BronchStop researcher (using the NHS SPINE portal in England ^26^, or equivalents in Scotland/Wales/Northern Ireland) if they are unable to do so.

Secondary outcomes include feeding support administered (nasogastric feeding, intravenous fluids), highest level of care afforded (high-dependency unit [HDU] or paediatric intensive care unit [PICU]), and respiratory support administered (low-flow oxygen, high-flow oxygen, continuous positive airway pressure [CPAP]) and invasive mechanical ventilation [IMV]).

If an infant was eligible for nirsevimab but did not receive this, mothers will be asked for comments on why this was the case by study researchers.

### Sample size and analysis plan

***Sample size***

Calculations were based on the precision of the VE estimated by the test-negative design, as recommended by the WHO, and implemented using their VE calculator ^27^. The latest figures for maternal RSVpreF vaccine coverage were 54.7% at the latest estimate in England ^28^ and 49.6% in Scotland ^13^; we therefore assumed 50% coverage for the purpose of these calculations. Assuming, based on MATISSE trial and real-world data from Argentina, that VE for infants aged 0–6 months is 10–15% lower than VE for those aged 0–3 months, we estimated the true VE for RSV-associated bronchiolitis/LRTI hospitalisation among infants from birth through 6 months to be 60% (compared with 72% in our initial study, where the median age of recruited case infants was 1.4 months). To achieve a precision width of 40% (+/-20%) for this VE estimate, the study would need to recruit 148 RSV-associated hospitalisations, with 1:1 matching with test-negative controls. This is an achievable prediction for a minimum target, for an unadjusted analysis. However, depending on the ratio of cases:controls that are found this season we may need a greater or smaller sample size to allow confident characterisation of some of our adjusting variables.

We also conducted sample size calculations for protection against PICU admissions and for nirsevimab to protect against RSV-associated bronchiolitis/LRTI hospitalisation. However, assuming an RSVpreF effectiveness of 65% in preventing PICU admission (as observed in the first season of the BronchStop study), we would need to recruit 120 PICU admissions, which is beyond the scope of this study. Equally, assuming 80% uptake of nirsevimab, and protection against hospitalisation of 68% for preterm infants (unpublished data from meta-analysis in press), 114 nirsevimab-eligible cases and 114 nirsevimab-eligible controls would be required, which was again judged to be beyond the scope of this study.

***Statistical analysis***

The primary effectiveness of maternal RSVpreF vaccination against RSV-associated hospitalisation in infants will be estimated using conditional logistic regression. Vaccine effectiveness will be calculated using the following equation: effectiveness = 100% x (1- adjusted odds ratio).

**Figure 1.**
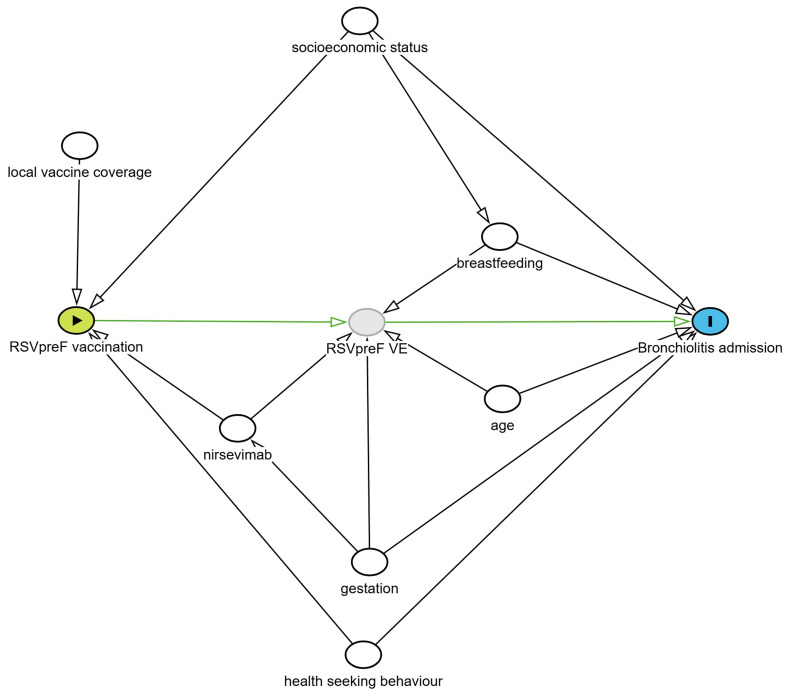
Directed acyclic graph (DAG) to identify potential confounders for season 2 of the BronchStop study. A test negative design was chosen to account for potential biases in health seeking behaviour. Based on this DAG, the initial analysis model will include geographic site, month of admission, gestation, socioeconomic status, age at admission and breastfeeding status. To account for the marked seasonality of RSV, as a sensitivity analysis we will perform an analysis including the number of month post-birth spent in the RSV season as a variable.

Identification of potential confounders was based on the use of a directed acyclic graph (DAG) ^17^ ( Figure 1). Based on this DAG, the initial analysis model will include geographic site, month of admission, gestation, socioeconomic status, age at admission and breastfeeding. Adjustment by site is used to allow for geographical differences in maternal vaccine uptake.

To look for evidence of confounding or other unmeasured sources of bias due to our analysis method we will also calculate the effectiveness of maternal pertussis vaccination against admission with RSV bronchiolitis using the same adjusting factors.

Demographic variables for cases and controls will be compared using a Wilcoxon rank sum test, a Pearson’s Chi-squared test or a Fisher’s exact test. Clinical outcomes in maternally vaccinated versus unvaccinated RSV positive cases will be compared using a Wilcoxon rank sum test, Pearson’s Chi-squared test or a Fisher’s exact test. The secondary analyses looking at VEs in different scenarios will use the same adjusting factors as for the primary analysis.

All statistical tests will be two-sided, and a P value of less than 0.05 considered to indicate statistical significance; p-values will not be adjusted for multiplicity. Confidence intervals will be calculated as 95% unless otherwise stated. The widths of the confidence intervals will not be adjusted for multiplicity and should therefore not be used in place of hypothesis testing. Statistical analyses will be carried out with the use of R software, version 4.5.2 (R Foundation for Statistical Computing).

### Data collection

Data will be entered using the validated online data entry software REDCap (Research Electronic Data Capture tools ^29^) using the clinical report forms provided in Supplementary File 1. This software (REDCap) is hosted on the Queen’s University Belfast server (data processor), under an information sharing agreement with the University Hospitals of Leicester NHS Trust (data controller). Data will be entered by participating sites, processed and securely stored using the Queen’s University Belfast REDCap.

***Sampling and laboratory testing***

All routinely collected samples for consented patients will be stored at a temperature of less than minus 20 degrees Celsius for subsequent testing: WGS for RSV positive samples, and rRT-PCR testing for all samples, RSV-positive and RSV-negative.

### Data management

Data will be entered using the validated online data entry software REDCap (Research Electronic Data Capture tools) following the clinical report forms provided in the appendix (Supplementary File 1). This software (REDCap) is hosted on the Queen’s University Belfast secure server. All research data reside within the hosting institution. The study Sponsor Organisation (University Hospitals of Leicester NHS Trust) will be the Data Controller throughout, and Queen’s University Belfast will be a data Processor for the duration of data entry and cleaning. REDCap uses a granular security model so that users can only review the data they have been explicitly authorised to access. REDCap also provides a comprehensive log/audit feature that records all individual changes with a date/time stamp and a change owner.

### Ethics and dissemination

The BronchStop study for the 2024–2025 season was submitted for Integrated Research Application System (IRAS) approval with the University Hospitals of Leicester NHS Trust as Study Sponsor, IRAS ID 297802, and received a favourable opinion from the Research Ethics Committee (REC) on the 8th August 2024; updates to the protocol will be submitted to the same REC. Participants recruited to the study will be asked for informed consent to participate in the maternal survey and for researchers to access their vaccination records.

### Dissemination

Data will be presented in:

- A real-time study dashboard
- Data submissions to the regulatory authorities, public health agencies and local study teams, including JCVI and WHO
- A study preprint
- A peer reviewed scientific journal
- All published outputs will be shared with members of the PPI group involved as part of study design, and those participants who consented to have study outputs shared with them during their initial consent

## Conclusion

Within a rapid time frame, we will aim to have data on vaccine effectiveness of maternal RSVpreF vaccination, in a steady stay national vaccination programme, through to 6 months of age. Virological testing will allow us to understand viral pathogens other than RSV in the post-vaccination era, and a survey of pregnant women will allow us to understand reasons for incomplete vaccine uptake.

## Data Availability

As this is a study protocol no data are yet available. Study data will be stored on a REDCap server hosted by Queen’s University Belfast, United Kingdom (See Data management system). Anonymised, aggregate data will be shared with interested parties upon reasonable request following approval from the sponsor institution (UHL NHS Trust). The study questionnaires are available at https://dx.doi.org/10.7488/era/885. Data are available under the terms of the Creative Commons Attribution 4.0 International license (CC-BY 4.0).
