# Supplementary material for: BronchStop Study Protocol, Season 2
Source: NIHR Open Res. 2025 Dec 2;5:85. Originally published 2025 Sep 23. [Version 2] doi: 10.3310/nihropenres.14097.2 (PMC12595303; doi:10.3310/nihropenres.14097.2)
Supplement: Peer review history

# Reviewer report: Reviewer response for version 2

**DOI:** 10.3310/nihropenres.15435.r38620

**Published:** 2025-12-24

**Authors:** Christine Blauvelt

**Affiliations:** 1 University of California San Francisco, San Francisco, California, USA

No further comments at this time.

Is the study design appropriate for the research question?

Yes

Is the rationale for, and objectives of, the study clearly described?

Yes

Are sufficient details of the methods provided to allow replication by others?

Yes

Are the datasets clearly presented in a useable and accessible format?

Not applicable

Reviewer Expertise:

Obstetrics; maternal-fetal medicine; infectious diseases in pregnancy

I confirm that I have read this submission and believe that I have an appropriate level of expertise to confirm that it is of an acceptable scientific standard.

# Reviewer report: Reviewer response for version 1

**DOI:** 10.3310/nihropenres.15330.r37610

**Published:** 2025-11-08

**Authors:** Allen Nazareno

**Affiliations:** 1 University of the Philippines Los Baños, Laguna, Philippines

The study is a UK-wide, multi-centre, prospective test-negative case-control study evaluating the effectiveness of maternal RSV vaccination in preventing hospitalisations for RSV-associated respiratory infections in infants under six months. It also examines factors influencing vaccine uptake and conducts molecular analyses to understand residual causes of severe respiratory disease in the post-vaccination era.

 I would like to thank the authors for submitting this protocol paper. The study is methodologically sound and clearly articulated. I find the manuscript scientifically well-founded. I recommend acceptance as it is.

Is the study design appropriate for the research question?

Yes

Is the rationale for, and objectives of, the study clearly described?

Yes

Are sufficient details of the methods provided to allow replication by others?

Yes

Are the datasets clearly presented in a useable and accessible format?

Not applicable

Reviewer Expertise:

mathematical epidemiology  and health economic evaluation

I confirm that I have read this submission and believe that I have an appropriate level of expertise to confirm that it is of an acceptable scientific standard.

# Response

**Published:** 2025-11-26

**Authors:** Thomas Williams

We sincerely thank the reviewer for this positive assessment and endorsement of the study protocol.

# Reviewer report: Reviewer response for version 1

**DOI:** 10.3310/nihropenres.15330.r37825

**Published:** 2025-11-07

**Authors:** Christine Blauvelt

**Affiliations:** 1 University of California San Francisco, San Francisco, California, USA

**Overview: **This is a multi-center test-negative case control study aimed at estimating vaccine effectiveness (VE) of the maternal RSVpreF vaccine in preventing RSV-positive acute lower respiratory infection (ALRI) hospitalization among infants under six months of age. This is a timely and relevant study addressing critical gaps following implementation of maternal RSV vaccination program in the UK. Strengths of the study include rRT-PCR confirmation of respiratory viruses, use of qualitative surveys to understand attitudes and knowledge contributing to RSV vaccine and nirsevimab uptake, and multi-center design.

**Points for Consideration:** RSV circulation is highly seasonal in the UK, with activity typically occurring between October and February. Infants born at the end of this period would have minimal RSV exposure within their first six months of life, even though their mothers were eligible for RSV vaccination during pregnancy. Analyses should therefore consider the number of months post-birth during the active RSV season, rather than relying solely on the calendar month of hospital admission.

Is the study design appropriate for the research question?

Yes

Is the rationale for, and objectives of, the study clearly described?

Yes

Are sufficient details of the methods provided to allow replication by others?

Yes

Are the datasets clearly presented in a useable and accessible format?

Not applicable

Reviewer Expertise:

Obstetrics; maternal-fetal medicine; infectious diseases in pregnancy

I confirm that I have read this submission and believe that I have an appropriate level of expertise to confirm that it is of an acceptable scientific standard.

# Response

**Published:** 2025-11-26

**Authors:** Thomas Williams

**Comment**

*“RSV circulation is highly seasonal in the UK, with activity typically occurring between October and February. Infants born at the end of this period would have minimal RSV exposure within their first six months of life, even though their mothers were eligible for RSV vaccination during pregnancy. Analyses should therefore consider the number of months post-birth during the active RSV season, rather than relying solely on the calendar month of hospital admission.”*

**Response**

 Thank you for this important methodological consideration. Seasonality is certainly a concern for the analysis, and there are many different approaches that one might take to address this.

 Similar to our first year analysis, our plan is to match by month of attendance and include age as a factor in the analysis, as we believe protection will wane rapidly over the first 6 months. This means that groups will have broadly the same RSV exposure.

 However, including age in months since the start of the start of RSV season as a variable would work too, and if added as a factor would probably result in a very similar analysis. We have edited the protocol to highlight that we will conduct this as a sensitivity analysis, but feel it would be helpful to remain consistent with our previous study for the primary analysis.

**Added to the Methods:**

To account for the marked seasonality of RSV, as a sensitivity analysis we will perform an analysis including the number of month post-birth spent in the RSV season as a variable.

# Reviewer report: Reviewer response for version 1

**DOI:** 10.3310/nihropenres.15330.r37614

**Published:** 2025-10-25

**Authors:** Jonne Terstappen

**Affiliations:** 1 University Medical Center Utrecht, Utrecht, Netherlands Antilles

BronchStop Study Protocol, Season 2

 Summary: With interest I have read the protocol for the BronchStop study season 2 by Shaun O’Hagan and colleagues. The study follows up on the 1 ^st^ BronchStart and BronchStop study launched in 2021, which assessed maternal RSVpreF vaccine effectiveness, motivation for vaccine uptake, and molecular diversity of RSV cases in the United Kingdom. Vaccine coverage was relatively low (+/- 50%), vaccine effectiveness could only be calculated for young infant (< 5 months), and in most cases no viral pathogen was determined. The season 2 study fill to answer these data gaps as well as evaluate reasons for incomplete nirsevimab uptake in high-risk infants. The results of this study can inform (inter)national vaccine implementation strategies as well as potential interventions to increase vaccine uptake. I applaud the authors on their thorough methodology.

 My main comment is that a questionnaire is not the most suitable method for understanding vaccine hesitancy. The data will not be as saturated as interviews or focus group discussions and you introduce selection bias as opiniated mothers might be more likely to fill out the questionnaire. The authors should consider recruiting mothers for a focus group discussion or interviews to better understand vaccine hesitancy.

 Other comments

1. Why have the authors chosen to only include infants < 6 months rather than < 12 months?
2. The parent other than the mother, when suitable, could also be involved in the research question about vaccine decision and hesitation as they might influence decision making.

Is the study design appropriate for the research question?

Yes

Is the rationale for, and objectives of, the study clearly described?

Yes

Are sufficient details of the methods provided to allow replication by others?

Yes

Are the datasets clearly presented in a useable and accessible format?

Not applicable

Reviewer Expertise:

RSV research

I confirm that I have read this submission and believe that I have an appropriate level of expertise to confirm that it is of an acceptable scientific standard.

# Response

**Published:** 2025-11-26

**Authors:** Thomas Williams

**Comment 1**

***“*** *My main comment is that a questionnaire is not the most suitable method for understanding vaccine hesitancy. The data will not be as saturated as interviews or focus group discussions and you introduce selection bias as opinionated mothers might be more likely to fill out the questionnaire. The authors should consider recruiting mothers for a focus group discussion or interviews to better understand vaccine hesitancy.”*

**Response**

 We thank the reviewer for this valuable suggestion and agree that qualitative interviews offer deeper insights into parental decision-making related to vaccination. Regarding selection bias, the maternal survey is administered to all study participants, thereby minimising this concern.

 We are in the process of seeking ethical approval for a qualitative study involving semi-structured interviews to explore parental attitudes towards the currently available RSV prevention options for infants. The study, *“Understanding Parental Decision-Making in RSV Prevention: The BronchStop Qualitative Study”,* will use purposive sampling to recruit parents and caregivers through community channels and via the BronchStop study, ensuring a broad range of perspectives and diverse representation in relation to parental age, geographical location, socio-economic status, educational attainment and self-reported ethnicity.

 A previously approved Research Ethics Committee (REC) amendment to the BronchStop study permits mothers recruited to the main study, to indicate at the end of the participant survey whether they would be interested in taking part in a more in-depth interview. In response to the reviewer’s comment, we have now incorporated information about this proposed qualitative study into the protocol manuscript, as follows. 

**Added to the end of the ‘Participant Questionnaire’ section:**

Additionally, mothers completing the participant questionnaire will be asked whether they are willing to be contacted about participating in an in-depth interview for a proposed qualitative study (currently under ethical review), titled *“Understanding Parental Decision-Making in RSV Prevention: The BronchStop Qualitative Study.”* This study will explore and interpret parental attitudes towards the maternal RSVpreF (Abrysvo®) vaccine in pregnancy and the infant long-acting monoclonal antibody, nirsevimab (Beyfortus®), in order to identify perceived barriers and facilitators to uptake of both preventatives. As well as interviewing mothers, we will conduct purposive sampling to recruit parents and caregivers through community channels to ensure a broad range of perspectives are captured. The findings of this study will inform the development of tailored antenatal education and counselling resources, as well as supporting ongoing vaccine implementation efforts across the UK.

**Comment 2**

*“Why have the authors chosen to only include infants <6 months rather than <12 months?”*

**Response**

 We limited the study population to infants <6 months of age because transplacentally transferred maternal antibodies decline over time in the infant. Current evidence indicates passive immunity conferred through maternal vaccination provides greatest protection during the first six months of life. Beyond this period, antibody titres and corresponding protection wane substantially, with limited data to support sustained vaccine efficacy up to 12 months of age. ^1,2^ Including infants up to 12 months would require a substantially larger sample size in order to maintain the desired precision of ±20% around the vaccine effectiveness (VE) estimate; exceeding the anticipated recruitment capacity of this study. Furthermore, focusing on infants under six months old aligns with the duration of outcome follow-up undertaken in the MATISSE trial. ^3^

  

1. Banooni P, Gonik B, Epalza C, et al. Efficacy, immunogenicity, and safety of an investigational maternal respiratory syncytial virus prefusion F protein-based vaccine. *Clin Infect Dis*. 2025 Jan 29:ciaf033.
2. Proto A, Agliardi S, Pani A, et al. COVID-Vaccines in Pregnancy: Maternal and Neonatal Response over the First 9 Months after Delivery. *Biomolecules*. 2024 Apr 3;14(4):435.
3. Kampmann B, Madhi SA, Munjal I, et al. Bivalent Prefusion F Vaccine in Pregnancy to Prevent RSV Illness in Infants. *N Engl J Med*. 2023 Apr 20;388(16):1451-1464.

In response to the reviewer’s comment, we have added two additional sentences relating to limiting the study population to infants aged <6 months:

**Added to the end of the ‘Inclusion Criteria’ section:**

We will recruit infants aged less than 6 months because passive immunity conferred through maternal vaccination is highest early in life and wanes thereafter, with limited data supporting sustained protection in infants beyond 6 months of life. ^23,24^ Additionally, including older infants (up to 12 months) would require a substantially larger sample size to maintain the desired precision for the maternal VE estimate, exceeding the anticipated recruitment capacity of this study.

**Comment 3**

*“The parent other than the mother, when suitable, could also be involved in the research question about vaccine decisions and hesitation as they might influence decision making.”*

**Response**

 We agree vaccine decision-making is often shared between parents/caregivers. In recognition of this, we will be recruiting parents other than the mother to the aforementioned qualitative study; *“Understanding Parental Decision-Making in RSV Prevention: The BronchStop Qualitative Study”*.
